# Pregnancy‐Related Bone Mineral and Microarchitecture Changes in Women Aged 30 to 45 Years

**DOI:** 10.1002/jbmr.3998

**Published:** 2020-04-08

**Authors:** Mícheál Ó Breasail, Ann Prentice, Kate Ward

**Affiliations:** ^1^ MRC Nutrition and Bone Health Research Group University of Cambridge Cambridge UK; ^2^ MRC Lifecourse Epidemiology Unit, University of Southampton Southampton General Hospital Southampton UK

**Keywords:** ANALYSIS/QUANTITATION OF BONE, BONE QCT/μCT, EPIDEMIOLOGY, GENERAL POPULATION STUDIES, AGING

## Abstract

At birth, the neonatal skeleton contains 20 to 30 g calcium (Ca). It is hypothesized maternal bone mineral may be mobilized to support fetal skeletal development, although evidence of pregnancy‐induced mineral mobilization is limited. We recruited healthy pregnant (*n* = 53) and non‐pregnant non‐lactating (NPNL; *n* = 37) women aged 30 to 45 years (mean age 35.4 ± 3.8 years) and obtained peripheral quantitative computed tomography (pQCT) and high‐resolution pQCT (HR‐pQCT) scans from the tibia and radius at 14 to 16 and 34 to 36 weeks of pregnancy, with a similar scan interval for NPNL. Multiple linear regression models were used to assess group differences in change between baseline and follow‐up; differences are expressed as standard deviation scores (SDS) ± SEM. Decreases in volumetric bone mineral density (vBMD) outcomes were found in both groups; however, pregnancy‐related decreases for pQCT total and trabecular vBMD were −0.65 ± 0.22 SDS and −0.50 ± 0.23 SDS greater (*p* < .05). HR‐pQCT total and cortical vBMD decreased compared with NPNL by −0.49 ± 0.24 SDS and −0.67 ± 0.23 SDS, respectively; trabecular vBMD decreased in both groups to a similar magnitude. Pregnancy‐related changes in bone microarchitecture significantly exceeded NPNL change for trabecular number (0.47 ± 0.23 SDS), trabecular separation (−0.54 ± 0.24 SDS), cortical thickness (−1.01 ± 0.21 SDS), and cortical perimeter (0.78 ± 0.23 SDS). At the proximal radius, cortical vBMD and endosteal circumference increased by 0.50 ± 0.23 SDS and 0.46 ± 0.23 SDS, respectively, compared with NPNL, whereas cortical thickness decreased −0.50 ± 0.22 SDS. Pregnancy‐related decreases in total and compartment‐specific vBMD exceed age‐related change at the distal tibia. Changes at the radius were only evident with pQCT at the cortical‐rich proximal site and suggest endosteal resorption. Although the magnitude of these pregnancy‐related changes in the appendicular skeleton are small, if they reflect global changes across the skeleton at large, they may contribute substantially to the Ca requirements of the fetus. © 2020 Crown copyright. *Journal of Bone and Mineral Research* published by Wiley Periodicals LLC on behalf of American Society for Bone and Mineral Research (ASBMR). This article is published with the permission of the Controller of HMSO and the Queen's Printer for Scotland.

## Introduction

Reproduction places increased pressure on maternal mineral economy to supply essential nutrients during pregnancy. Fetal calcium (Ca) accretion increases from approximately 50 mg/d at 20 weeks to 330 mg/d at 35 weeks,^1^ and at birth a newborn skeleton contains 20 to 30 g Ca.^2^ Biological strategies allow the mother to meet these extra nutritional demands. These may include increases in mineral intake and physiological adaptations including increased gastrointestinal absorption, increased renal clearance, increased urinary excretion, and potentially bone mineral mobilization.^3^, ^4^, ^5^, ^6^, ^7^ Several studies have tried to quantify pregnancy‐induced bone loss but with conflicting results.^7^, ^8^, ^9^, ^10^, ^11^, ^12^, ^13^, ^14^, ^15^, ^16^, ^17^, ^18^, ^19^, ^20^ From the available data, pregnancy and lactation are not considered risk factors for osteoporosis,^6^ although as noted in a recent review, there may be permanent alterations in skeletal structure with implications for the metabolic and mechanical function of bone.^21^

A broad variety of techniques including circulating hormone levels,^22^, ^23^, ^24^, ^25^, ^26^, ^27^ bone turnover markers (BTM),^15^, ^28^, ^29^, ^30^, ^31^ bone histomorphology via bone biopsies,^32^, ^33^ quantitative ultrasound (QUS),^34^, ^35^ and circulating lead concentrations^36^ have been used. However, the few studies that have used X‐ray‐based techniques to explore pregnancy‐related skeletal changes have typically either obtained dual‐energy X‐ray absorptiometry (DXA) scans pre‐pregnancy and postpartum or scanned the forearm with DXA during pregnancy to quantify the changes in areal bone mineral density (aBMD). Both these approaches have limitations because comparing pre‐pregnancy and postpartum scans are confounded by the initiation of lactation, and forearm DXA cannot distinguish between cortical and trabecular bone. Peripheral quantitative computed tomography (pQCT) and high resolution (HR)‐pQCT overcome these limitations and involve a minimal amount of ionizing radiation. Despite this, to date, only two studies in pregnancy have been published using single‐slice pQCT^37^, ^38^ and none using HR‐pQCT.

Therefore, this study aimed to quantify the extent of pregnancy‐induced changes in compartmental (trabecular and cortical) volumetric BMD (vBMD) and bone mineral microarchitecture using pQCT and HR‐pQCT techniques between the early second (14 to 16 weeks) and the third trimester (34 to 36 weeks) in UK women aged 30 to 45 years. This age range was selected to focus on the groups where numbers of pregnancies have risen most rapidly in recent years.^39^ As age‐related changes in pQCT and HR‐pQCT bone outcome measures have been reported in non‐pregnant non‐lactating (NPNL) women in this age range,^40^, ^41^, ^42^ a control group of healthy NPNL women was followed over the same period. The primary hypothesis was that in pregnant women, there would be decreases in trabecular vBMD measured by pQCT and HR‐pQCT and changes in trabecular microarchitecture as measured by HR‐pQCT (ie, reduced trabecular thickness and reduced trabecular number). Secondary hypotheses were that there would be reduced cortical thickness and density in addition to endosteal resorption at the proximal radius and tibia by pQCT and distal radius and tibia by HRpQCT.

## Materials and Methods

### Recruitment

Women with a singleton pregnancy achieved by natural conception and aged 30 to 45 years were recruited between March 2017 and December 2018 at the Rosie Hospital Cambridge University Hospitals (CUH), Cambridge, UK. Pregnant women were approached at their 12‐week dating ultrasound scan by research midwives not actively involved in the participants' care. Pregnant women were also recruited from the local community through advertisements but only if they were under the medical care of the Rosie Hospital. Participants received a participant information sheet and were subsequently screened by a member of the research team via telephone interview. Visit 1 took place between 14 and 16 weeks of gestation, based on estimated gestational age from the 12‐week dating scan. A control group of healthy NPNL women in the same age range was recruited from the local community through posters and online advertisements and screened by telephone interview. Eligibility criteria for both groups were age 30 to 45 years, at least 3 months since cessation of lactation from previous pregnancy, no history of prolonged periods of amenorrhea, not taking medications known to alter bone or Ca metabolism, no history of conditions affecting bone or Ca metabolism, no history of prolonged immobilization or bed rest and the ability to give informed written consent. Before follow‐up, the continuing eligibility of pregnant participants was reassessed at 30 weeks’ gestation by the midwives by telephone. Participants who had serious pregnancy complications or who had moved from the area were not invited to attend the follow‐up visit.

Ethical approval was obtained from the East of England–Essex Research Ethics Committee (REC) (Project ID 199857) and R&D approval was obtained from Cambridge University Hospitals (reference number A094123). Informed written consent for all participants was acquired at the baseline visit.

### Anthropometry

Anthropometric measurements were made at each visit. Height (cm) was obtained without footwear to the nearest 1 mm using a wall‐mounted stadiometer (Seca GmbH, Hamburg, Germany). Weight (kg) was measured using a digital scale to the nearest 0.1 kg, while the participants wore light clothing without footwear (Seca GmbH, Hamburg, Germany). Subsequently body mass index (BMI, kg/m^2^) was calculated. To determine the appropriate scan sites for pQCT, both forearm and lower‐leg length were measured to the nearest 1 mm using a metal rule: Tibia length was measured from the distal edge of the medial malleolus to the tibial plateau; ulna length was recorded as the distance from the olecranon to the ulnar styloid process.

### Bone densitometry

Peripheral QCT (XCT 2000L, Stratec Medizintechnik, Pforzheim, Germany) and HR‐pQCT (Scanco XtremeCTI, Scanco Medical AG, Bruttisellen, Switzerland) scans at both baseline and follow‐up were acquired at the nondominant radius and tibia unless there had been a previous fracture or injury necessitating the immobilization of the limb, when the dominant limb was scanned. The total effective dose to the participant was estimated to be <18.4 μSv.

### pQCT

pQCT scans were obtained, with a voxel size of 0.5 × 0.5 mm and slice thickness of 2 mm, at the radius (at 4% and 33% of the limb length proximal to the distal endplate) and tibia (at 4% and 38% of the limb length proximal to the distal endplate). CT scan speed was 30 mm/s and scout view scan speed was 40 mm/s. pQCT scans were processed using the manufacturer's software (Stratec XCT version 6.2). At distal 4% sites, CALCBD analysis was used to calculate total cross‐sectional area (CSA) and total and trabecular vBMD. CALCBD contour mode 1 (ie, threshold algorithm) was used to exclude pixels in the defined region of interest (ROI) that fell below the default threshold of 180 mg/cm^3^; peel mode 1 (ie, concentric peel) peeled away the outer 55% of the total CSA of the bone, leaving an inner 45% CSA considered to be purely trabecular. At proximal cortical‐rich sites, CORTBD was used to define cortical vBMD and area. The algorithm removes all voxels within the ROI that have an attenuation coefficient below the threshold. The default threshold of 710 mg/cm^3^ was used with separation mode 1. Total CSA was defined at proximal sites at a threshold of 280 mg/cm^3^. Cortical thickness, endosteal circumference, and periosteal circumference were calculated using a circular ring model. Scans were qualitatively graded by visual inspection to assess their suitability for longitudinal analysis: Scan slices with excessive movement or other artifacts and scout views that did not match longitudinally were excluded. Calibration of the XCT 2000 L system was performed on a routine basis with the manufacturer's phantom: Daily QA scans and weekly QC scans were performed throughout the study period to test scanner performance. At our research site, the precision for pQCT has been calculated to be 0.4% to 1.6% at the tibia and 0.9% to 4.3% at the radius by performing two repeat scans on 30 participants.

### High‐resolution pQCT

HR‐pQCT scans were obtained with the manufacturer's guidelines as described by Boutroy and colleagues.^(43)^ Briefly, before the initiation of each scan, antero‐posterior 2D scout views were performed to determine the scan region with a reference line manually placed at the endplate of the radius or tibia. After this, a stack of parallel CT slices was acquired using a 2D detector array; the first slice was obtained 9.5 mm and 22.5 mm proximal to the reference line for the radius and tibia, respectively. All scans were acquired by trained technicians using the standard positioning techniques.^43^ The quality of the HR‐pQCT scans of the distal radius and tibia was assessed using a five‐grade scale.^44^ Images with significant motion artifacts causing blurring and discontinuities in the cortical shell were excluded. HR‐pQCT scans were selected for longitudinal analysis if the matched‐common region between baseline and follow‐up scans was ≥80%. The matching of scans used the standard methodology from Scanco.^43^ All HR‐pQCT outcome measures were obtained using the manufacturer's standard evaluation.^43^ In contrast to conventional pQCT, trabecular vBMD was derived by system‐defined boundaries based on thresholding without manual correction. Coefficients of variation for HR‐pQCT from the manufacturer's standard analysis have been reported as 0.1% to 0.3% and 1.1% to 4.8% for density parameters and 0.4% to 5.2% and 2.7% to 12.7% bone microarchitecture at the tibia and radius, respectively.^45^

### Fracture history and lifestyle questionnaire

Additional information relevant to bone health, including parity, smoking, and hormonal contraceptive use, was obtained by questionnaire.

### Statistical approach

#### Sample size

In the absence of published values of pregnancy‐related changes using pQCT or HR‐pQCT, DXA‐measured size‐adjusted bone mineral content (BMC) at the lumber spine between pre‐pregnancy and 2 weeks postpartum of 2.6% ± 4.2 SEM^7^ was used for the sample size calculation. This determined that a sample size of 35 participants would be required to detect within‐group change in BMD from the first set of measurements (baseline, early second trimester of pregnancy) to the second measurements (third trimester of pregnancy) with a power of 0.9 and significance of 0.05. An equal number of NPNL participants would be recruited to determine natural‐age related changes.

#### Data analysis

All analyses were performed using R version 3.3.2 (R Foundation for Statistical Computing, Vienna, Austria; https://www.r-project.org/) with dplyr (version 0.8.3) for data manipulation.^46^ Baseline descriptive data are presented as mean (SD), except for parity, expressed as median (IQR), and for the number of participants who had ever taken contraceptives and/or ever smoked, given as count and percentage of the group (*n* [%]). Differences between pregnant and NPNL women at baseline were tested for using Student's *t* tests or Mann–Whitney *U* tests as appropriate. Unadjusted change between baseline and second visit for each variable of interest was calculated in absolute units and is reported as % change (± SD) and one‐sided *t* tests were used to explore whether within‐group change was statistically significant.

Change in bone variables was characterized by estimating linear regression models for outcome measures at follow‐up on each outcome measure at baseline; standardized residuals from these models function as Twisk's recommended measure of “residual change” when data from only two time points are available and yield a measure of change that is independent of baseline level.^47^ Using this conditional change approach also has the advantage of minimizing any effects in statistical models of the high correlation between baseline and follow‐up measures.

Multiple linear regression models were used to assess group differences in change between baseline and follow‐up using these standardized residuals. Covariates included in the initial models were group, baseline height, baseline weight, age, parity (0/≥1), smoking history (yes/no), and previous use of contraceptives (yes/no). The MASS package in R was used to select a model of best fit for each bone outcome measure. Initially backwards regression was performed (ie, the least significant covariate being removed first and then sequentially) and all models were then compared and the model with lowest Akaike information criterion (AIC) was selected in each case;^48^ in these models of best fit, confounders remained in the model irrespective of significance. The reason for selecting this analysis approach was because of multiple collinearity of the covariates when all were simultaneously included. Group was always included regardless of significance. In the models, the group coefficients are reported as the difference in SDS ± SEM, representing the between‐group difference in the within‐group change between baseline and follow‐up.

## Results

Eighty‐seven percent of pregnant (*n* = 46/53) participants and all NPNL (*n* = 37) participants were scanned at follow‐up. Mean (SD) estimated gestational age at baseline was 15.5 (0.9) weeks from the dating scan. Time to follow‐up for pregnant women was 20.1 (2.0) weeks (visit 2 mean gestational age 35.8 [2.1] weeks), and NPNL time to follow‐up did not differ significantly (21.6 [1.7] weeks). The only significant between‐group baseline difference in participant characteristics (Table 1) was parity, as controls had a greater median parity (*p* = .02). Tables 2 and 3 present bone data from both visits and percentage change from baseline for the tibia and radius, respectively. At baseline, significant between‐group differences in bone outcome measures were only found for tibial trabecular number, which was 9% lower in pregnant women compared with NPNL (1.98 [0.27] versus 2.13 [0.27] mm^−1^, *p* < .05, Table 2), and trabecular thickness, which was 11% higher compared with NPNL (0.07 [0.01] versus 0.07 [0.01] mm, *p* < .05, Table 2). Within‐group changes are presented in Tables 2 and 3; in general, the magnitude of change for bone outcomes in the pregnant group exceeded those of the NPNL group.

**Table 1:** Descriptive Statistics for Pregnant and Non‐Pregnant Non‐Lactating Women (NPNL)

	Pregnant (*n* = 46)	NPNL (*n* = 37)
Age (years)	35.0 (3.7)	36.0 (4.0)
Weight at visit 1 (kg)	68.2 (13.6)	70.4 (13.7)
Height (cm)	165.3 (5.9)	164.2 (7.2)
BMI (m/kg^2^)	24.9 (4.5)	26.2 (5.2)
Parity, median [IQR]	1 [0, 2]	2 [0, 4]^a^
Contraceptive use (ever), *n* (%)	43 (93)	34 (92)
Smoking (ever), *n* (%)	18 (39)	13 (35)
Weeks pregnant at visit 1	15.2 (2)	—
Weeks pregnant at visit 2	35.4 (2.2)	—
Weeks between visits	20.1 (2.0)	21.7 (1.7)

BMI = body mass index.

a *p* < .05 Mann–Whitney *U* test between groups at baseline.

**Table 2:** Descriptive Statistics at Visits 1 and 2 and Mean Difference Between Visits Expressed as Percentages (Mean ± SD) for pQCT‐ and HR‐pQCT‐Measured Bone Density, Microarchitecture, and Geometry Parameters at the Tibia in Pregnant and Non‐Pregnant Non‐Lactating Women (NPNL)

	Pregnant			NPNL		
	Visit 1	Visit 2	∆%	Visit 1	Visit 2	∆%
HR‐pQCT distal tibia		*n* = 33			*n* = 32	
Total vBMD (mg/cm^3^)	317.43 (68.09)	312.73 (66.02)	**−1.39 (1.73)** c	315.26 (53.64)	313.77 (53.70)	**−0.48 (1.00)** b
Cortical vBMD (mg/cm^3^)	916.26 (38.81)	908.84 (39.13)	**−0.81 (1.37)** b	921.45 (33.66)	920.60 (33.21)	−0.08 (0.70)
Trabecular vBMD (mg/cm^3^)	174.68 (41.33)	171.90 (40.26)	**−1.48 (1.97)** c	165.53 (38.02)	162.98 (37.70)	**−1.56 (1.48)** c
Trabecular number (mm^−1^)	1.98 (0.27)	2.07 (0.30)	**4.58 (7.61)** b	2.13 (0.27)	2.12 (0.31)	−0.29 (7.18)
Trabecular thickness (mm)	0.07 (0.01)	0.07 (0.01)	**−5.35 (6.68)** c	0.07 (0.01)	0.06 (0.01)	−0.78 (6.92)
Trabecular separation (mm)	0.44 (0.08)	0.43 (0.08)	**−3.66 (6.75)** c	0.41 (0.06)	0.42 (0.07)	1.10 (7.43)
Cortical thickness (mm)	1.21 (0.29)	1.20 (0.28)	**−1.21 (1.75)** b	1.25 (0.22)	1.26 (0.22)	**0.78 (1.06)** c
Cortical perimeter (mm)	99.6 (8.3)	99.80 (8.31)	**0.25 (0.25)** c	99.91 (7.16)	99.93 (7.15)	0.03 (0.24)
pQCT 4% tibia		*n* = 43			*n* = 34	
Total vBMD (mg/cm^3^)	306.25 (47.42)	303.18 (46.14)	**−0.95 (1.56)** c	301.57 (40.36)	301.39 (40.77)	−0.07 (1.04)
Trabecular vBMD (mg/cm^3^)	227.91 (44.01)	225.75 (44.15)	**−0.98 (1.65)** c	219.59 (39.15)	218.70 (39.14)	**−0.42 (1.08)** a
Total CSA (mm^2^)	980.36 (115.47)	975.63 (112.06)	**−0.44 (1.40)** a	989.76 (111.06)	989.08 (108.15)	−0.03 (1.09)
pQCT 38% tibia		*n* = 42			*n* = 35	
Total CSA (mm^2^)	378.45 (43.34)	379.37 (43.93)	0.24 (1.02)	392.90 (45.52)	394.16 (45.56)	0.32 (1.08)
Cortical vBMD (mg/cm^3^)	1172.08 (19.34)	1170.56 (18.77)	−0.13 (0.51)	1169.09 (24.25)	1169.23 (23.04)	0.02 (0.40)
Cortical BMC (mg/mm)	315.44 (38.98)	315.18 (39.11)	−0.07 (1.35)	324.76 (40.87)	324.59 (40.97)	−0.05 (1.03)
Cortical CSA (mm^2^)	269.21 (33.59)	269.36 (33.98)	0.06 (1.39)	277.81 (34.70)	277.60 (34.54)	−0.07 (1.26)
Cortical thickness (mm)	4.84 (0.44)	4.82 (0.45)	−0.49 (1.65)	4.91 (0.55)	4.88 (0.55)	**−0.69 (1.58)** a
Periosteal circumference (mm)	70.75 (4.18)	70.98 (4.16)	**0.33 (0.86)** a	72.09 (4.59)	72.35 (4.58)	**0.35 (0.73)** b
Endosteal circumference (mm)	40.34 (3.52)	40.72 (3.52)	**0.97 (2.19)** b	41.24 (5.20)	41.70 (5.25)	**1.14 (2.06)** b

Bold indicates a statistically significant finding i.e. (*p* < .05).

vBMD = volumetric bone mineral density; CSA = cross‐sectional area.

a *p* < .05.

b *p* < .01.

c *p* < .001 single‐sided *t* test versus 0.

**Table 3:** Descriptive Statistics at Visits 1 and 2 and Mean Difference Between Visits Expressed as Percentages (Mean ± SD) for pQCT‐ and HR‐pQCT‐Measured Bone Density, Microarchitecture, and Geometry Parameters at the Radius in Pregnant and Non‐Pregnant Non‐Lactating Women (NPNL)

	Pregnant			NPNL		
	Visit 1	Visit 2	∆%	Visit 1	Visit 2	∆%
HR‐pQCT distal radius		*n* = 33			*n* = 27	
Total vBMD (mg/cm^3^)	334.20 (58.74)	331.32 (60.12)	**−0.94 (2.04)** a	334.75 (63.13)	331.61 (62.23)	**−0.90 (2.31)** a
Cortical vBMD (mg/cm^3^)	920.46 (42.47)	916.15 (43.84)	**−0.47 (1.05)** a	916.35 (49.22)	912.91 (49.78)	−0.37 (1.12)
Trabecular vBMD (mg/cm^3^)	153.28 (39.86)	151.11 (40.09)	**−1.54 (2.07)** c	155.10 (32.80)	152.57 (33.02)	**−1.74 (1.49)** c
Trabecular number (mm^−1^)	1.95 (0.34)	1.96 (0.33)	1.27 (10.99)	2.02 (0.23)	2.06 (0.27)	2.06 (10.11)
Trabecular thickness (mm)	0.07 (0.01)	0.06 (0.01)	−1.79 (9.76)	0.06 (0.01)	0.06 (0.012)	**−2.93 (8.58)** a
Trabecular separation (mm)	0.46 (0.10)	0.46 (0.10)	−0.012 (9.93)	0.44 (0.06)	0.43 (0.069)	−0.86 (9.61)
Cortical thickness (mm)	0.82 (0.14)	0.82 (0.15)	−0.75 (3.40)	0.81 (0.16)	0.81 (0.15)	0.50 (3.98)
Cortical perimeter (mm)	66.68 (5.80)	66.75 (6.00)	0.07 (0.52)	66.74 (5.35)	66.65 (5.35)	−0.16 (0.56)
pQCT 4% radius		*n* = 40			*n* = 31	
Total vBMD (mg/cm^3^)	314.64 (48.69)	313.37 (45.96)	−0.12 (5.33)	323.20 (51.77)	326.05 (52.52)	0.96 (4.46)
Trabecular vBMD (mg/cm^3^)	176.45 (32.83)	176.51 (32.36)	0.16 (3.04)	180.41 (38.03)	181.17 (37.44)	0.52 (2.38)
Total CSA (mm^2^)	335.80 (45.41)	336.14 (45.95)	0.18 (4.31)	328.69 (45.69)	328.25 (46.64)	−0.09 (4.06)
pQCT 33% radius		*n* = 41			*n* = 35	
Total CSA (mm^2^)	98.05 (12.56)	98.13 (12.54)	0.10 (1.30)	99.43 (12.33)	98.92 (11.95)	−0.46 (1.54)
Cortical vBMD (mg/cm^3^)	1217.49 (15.81)	1220.46 (13.32)	0.25 (1.02)	1218.13 (17.45)	1216.82 (17.34)	−0.11 (0.64)
Cortical BMC (mg/mm)	87.66 (10.31)	88.06 (10.37)	**0.47 (1.18)** a	88.88 (9.37)	89.08 (9.41)	0.22 (1.11)
Cortical CSA (mm^2^)	72.02 (8.55)	72.17 (8.59)	0.23 (1.65)	72.96 (7.63)	73.21 (7.72)	0.33 (1.41)
Cortical thickness (mm)	2.55 (0.22)	2.56 (0.22)	0.43 (2.79)	2.54 (0.21)	2.58 (0.22)	**1.63 (2.96)** b
Periosteal circumference (mm)	36.21 (2.34)	36.19 (2.32)	−0.04 (1.50)	36.68 (2.08)	36.46 (2.06)	**−0.61 (1.49)** a
Endosteal circumference (mm)	20.20 (2.30)	20.08 (2.29)	−0.34 (4.35)	20.71 (2.10)	20.22 (2.12)	**−2.26 (4.62)** b

Bold indicates a statistically significant finding i.e. (*p* < .05).

vBMD = volumetric bone mineral density; CSA = cross sectional area; BMC = bone mineral content.

a *p* < .05.

b *p* < .01.

c *p* < .001 single‐sided *t* test versus 0.

### Changes at the tibia

**Figure 1:**
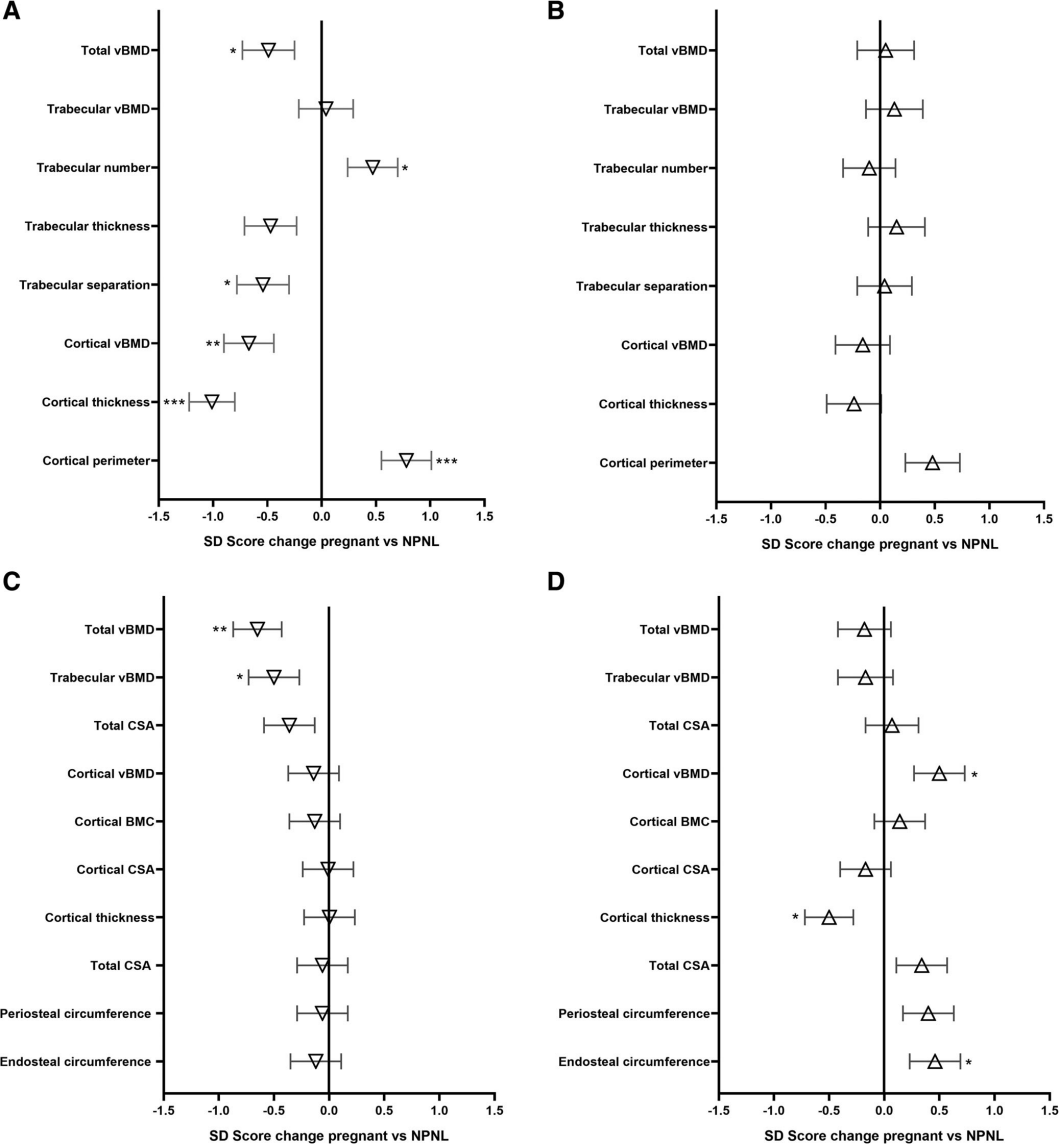
Regression coefficient and standard error of the mean for HR‐pQCT ([*A*] tibia; [*B*] radius) and pQCT ([*C*] tibia; [*D*] radius) parameters. All parameters expressed as standardized deviation scores (SDS ± SEM).

In pregnant women, total and cortical vBMD (HR‐pQCT) at the distal tibia decreased relative to the NPNL group by 0.49 (0.24) SDS and 0.67 (0.23) SDS, respectively (*p* = .04 and *p* < .01) (Fig. 1, Table 4). Trabecular bone microarchitecture differed significantly between groups: Trabecular number increased by 0.48 (0.23) SDS in pregnant woman compared with NPNL, whereas trabecular separation 0.54 (0.24) SDS decreased in pregnancy (*p* = .02, *p* < .01, respectively). Trabecular thickness also decreased in pregnant women relative to controls, though this was not statistically significant (*p* = .06). HR‐pQCT cortical thickness decreased by −1.01 (0.21) SDS, whereas cortical perimeter increased by 0.78 (0.23) SDS (both *p* < .001). For pQCT at the distal tibia, significant decreases in total and trabecular vBMD of 0.65 (0.22) SDS and 0.50 (0.23) SDS, respectively, were found for pregnant compared with controls (*p* < .01 and *p* < .05, respectively). In contrast, no significant between‐group differences were found at the cortical‐rich 38% proximal tibia site.

**Table 4:** Multiple Regression Models of Change in pQCT and HR‐pQCT Bone Outcome Measures at the Distal and Proximal Tibia (Data Reported as SDS ± SEM)

Dependent variable	Tibia		Radius	
	Group β (SD score ± SEM)	Covariates in model of best fit	Group β (SD score ± SEM)	Covariates in model of best fit
HR‐pQCT	*n* = 65		*n* = 60	
Total vBMD	**−0.49 ± 0.24** a	**group** a, **age** a, contraceptives	0.05 ± 0.26	group, **age** a
Trabecular vBMD	0.04 ± 0.25	group, age	0.13 ± 0.26	group
Trabecular number	**0.47 ± 0.23** a	**group** a, **weight** a, **parity** a	−0.10 ± 0.24	group, **weight** a, **parity** a
Trabecular thickness	−0.47 ± 0.24	group, weight, parity	0.15 ± 0.26	group, weight, parity
Trabecular separation	**−0.54 ± 0.24** a	**group** a, weight, parity	0.04 ± 0.25	group, **weight** a, **parity** a
Cortical vBMD	**−0.67 ± 0.23** b	**group** b, **weight** a	−0.16 ± 0.25	group, **weight** a, parity
Cortical thickness	**−1.01 ± 0.21** c	**group** c, age	−0.24 ± 0.25	group, **age** a
Cortical perimeter	**0.78 ± 0.23** c	**group** c, parity	−0.60 ± 0.22	group
pQCT 4%	*n* = 77		*n* = 71	
Total vBMD	**−0.65 ± 0.22** b	**group** b	−0.18 ± 0.24	group, height, parity, contraceptives (ever)
Trabecular vBMD	**−0.50 ± 0.23** a	**group** a, weight, parity, ever smoked	−0.17 ± 0.25	group, weight, parity, smoked (ever)
Total CSA	−0.36 ± 0.23	group	0.07 ± 0.24	group
pQCT 33/38%	*n* = 77		*n* = 76	
Cortical vBMD	−0.14 ± 0.23	group, age, height, contraceptives (ever)	**0.50 ± 0.23** a	**group** a, parity, smoked (ever)
Cortical BMC	−0.13 ± 0.23	group, weight, **height** a	0.14 ± 0.23	group, **parity** a
Cortical CSA	−0.01 ± 0.23	group, weight, **height** a	−0.17 ± 0.23	group, weight, smoked (ever), **parity** b
Cortical thickness	0.003 ± 0.23	group, **height** a, contraceptives (ever)	**−0.50 ± 0.22** a	**group** a, **age** a
Total CSA	−0.06 ± 0.23	group, smoked (ever)	0.34 ± 0.23	group, **parity** a
Periosteal circumference	−0.06 ± 0.23	group	0.40 ± 0.23	group, age
Endosteal circumference	−0.12 ± 0.23	group	**0.46 ± 0.23** a	**group** a, **age** a

Models were run with group, baseline height, weight, age, parity, smoking (ever), and contraceptives (ever) with the model of best fit selected by comparing the model AIC values. Bold indicates a statistically significant finding i.e. (*p* < .05).

β = beta coefficient; ∆SDS = standard deviation score; vBMD = volumetric bone mineral density; BMC = bone mineral content; CSA = cross‐sectional area.

a *p* < .05.

b *p* < .01.

c *p* < .001.

### Changes at the radius

At the distal radius, no significant difference in any HR‐pQCT or pQCT bone outcome was found between pregnant women and controls. At the 33% proximal radius, cortical vBMD and endosteal circumference increased in the pregnant group by 0.50 (0.22) SDS and 0.46 (0.23) SDS, respectively, compared with controls (both *p* < .05, Table 4). Cortical thickness decreased by 0.50 (0.23) SDS in pregnant women compared with NPNL (*p* < .05).

## Discussion

This study presents novel evidence of compartment‐specific changes in maternal bone mineral density, microarchitecture, geometry, and distribution during pregnancy in women aged 30 to 45 years old. These exceeded the natural age‐related changes that were observed in our control group, although in keeping with previous studies using pQCT techniques in the appendicular skeleton, our control group did have small decreases in several vBMD and related outcomes suggesting a midlife decline from peak bone mass.^40^ Given that the bone‐related changes found in pregnant women occur over and above this natural age‐related decline, the long‐term impact of pregnancy‐related mineral mobilization in women in this age range may be modulated by their ability to restore mobilized mineral post‐lactation and before the onset of menopause.

The magnitude of pregnancy‐related changes were relatively modest across our follow‐up period of approximately 20 weeks but were broadly consistent between two distinct scanning technologies. The decision not to scan beyond 36 weeks’ gestation was a pragmatic one, and it is entirely conceivable that total mineral mobilization across the entirety of pregnancy would exceed those presented here.

It is well established that fetal calcium accrual is greatest in late gestation, and DXA studies with scans from preconception to within 2 weeks postpartum have previously posited that pregnancy could lead to the mobilization of approximately 2% of maternal skeletal calcium. Our data would seem to support this, and although we only have peripheral measurements, it is likely (given what we know of lactation‐induced mobilization) that axial mobilization would be greater. What our data do suggest is that the maternal skeleton is likely to be an important source of calcium for the developing fetus.

### Tibia

Pregnancy‐related change was most evident at the distal tibia where total vBMD (HR‐pQCT and pQCT) decreased relative to controls. However, total vBMD includes both trabecular and cortical compartments and more nuance can be obtained from interpreting compartment vBMD. Compartment‐specific changes in vBMD were found with HR‐pQCT (cortical vBMD) and pQCT (trabecular vBMD). Decreases in HR‐pQCT cortical vBMD at the distal tibia were accompanied by decreases in cortical thickness but also an increase in cortical perimeter, perhaps as a compensatory adaptation. Despite no HR‐pQCT trabecular vBMD group effect, changes in tibia trabecular microarchitecture were observed with trabecular number increased and trabecular separation decreased relative to controls. Although trabecular thickness also decreased in pregnant women versus NPNL, it was not statistically significant (*p* = .06). In absolute terms, within‐group decreases in HR‐pQCT trabecular vBMD were of similar magnitude in both groups, while decreases in pQCT trabecular vBMD were much greater in pregnant women. As such, it may be that this discrepancy between pregnancy‐related change in pQCT and HR‐pQCT trabecular vBMD outcomes is the result of the slightly different relative compositions of the two scan sites imaged because the larger HR‐pQCT volume will have contained a greater proportion of trabecular bone than the pQCT scan slice. Also, the pQCT trabecular region focuses on the inner 45% of bone, which may be preferentially mobilized to limit the effects of bone loss on the mechanical integrity of bone.^21^

Pregnancy‐related changes in trabecular microarchitecture without trabecular vBMD (HR‐pQCT) changes require interpretation in the context of changes observed in the cortical compartment (ie, decreased cortical vBMD and thickness). First, it is possible that trabecular microarchitecture changes could be indicating cortical bone mobilization at the trabecular‐cortical interface; pregnancy‐related endosteal resorption leading to cortical thinning coupled with increasing cortical porosity could “trabecularize” the endocortical surface. This could possibly lead to pores close to the inter‐compartment junction being detected as part of the trabecular compartment, thereby increasing the mean trabecular number but decreasing the mean trabecular thickness. To further investigate that this lack of significant change in the trabecular compartment was not due to intracompartment reorganization, Dinn (trabecular vBMD of the inner 60% of the trabecular area), Dmeta (remaining 40%), and ratio of Dinn/Dmeta were also subsequently modeled but showed no pregnancy‐related change. Second, the finding of microarchitectural changes in the trabecular compartment may relate to the fact the HR‐pQCT voxel size is 82 μm, and although the largest trabeculae are ~100 μm, many may be considerably smaller. It may be possible that at baseline, trabeculae that were very close together were measured as one large trabecula, and that, as mineral was resorbed, these trabeculae were detected at follow‐up, leading to an increase in measured trabecular number. This would result in a lower mean trabecular separation and trabecular thickness.

### Radius

There was no evidence of pregnancy‐induced mineral mobilization at the distal radius, which may suggest that in this population, bone mineral is conserved during pregnancy at this site. The majority of single photon absorptiometry, dual photon absorptiometry, and DXA studies during pregnancy have found no pregnancy‐related changes in distal radius aBMD, and while one previous pQCT study reported changes in radius pQCT trabecular vBMD,^37^ a more recent pQCT study has not.^38^ In contrast, pregnancy‐related changes in vBMD, geometry, and distribution were found at the cortical‐rich proximal radius. From the models, a significant increase in cortical vBMD was observed, although given the changes reported in cortical thickness and endosteal circumference, this could be an artifact of bone mineral mobilization: As mineral was resorbed from the endocortical surface, the mean density of voxels nearest the medullary cavity would decrease below the 710 mg/cm^3^ threshold at which cortical bone is measured artificially, increasing the remaining voxels of the cortex. Decreasing cortical thickness and increasing endosteal circumference suggest mobilization from the endocortical surface supporting the hypothesis of Garn and others that bone mineral accrued during growth at the endocortical surface may act as a reservoir for use during reproduction.^49^

### Between‐site differences in change

The pattern of pregnancy‐related change between the distal tibia and radius is intriguing. The major difference between these two sites is that the distal tibia is a load‐bearing site in contrast to the distal radius. Previous studies using DXA conducted by our group have observed the conservation of bone mineral at the forearm during lactation while mineral was lost from the weight‐bearing hip and lumbar spine.^7^, ^50^ Most studies in the literature where the distal forearm has been scanned with DXA show no significant bone loss during reproduction. Animal data suggest that the skeletal system responds to increased calcium requirements during pregnancy and lactation by selectively degrading bone from trabecular‐rich sites where the bone may have a more metabolic rather than mechanical function.^21^ As such, the loss would have least impact on the mechanical competence of bone. The sites where this occurs are the more load‐bearing regions, where cortical bone is greatest and may adapt, postpartum, to maintain skeletal integrity.^21^ At present, human data are few, though the differing pattern of change reported in the current study indicates some preferential bone loss at the tibia compared with the radius. However, it is possible that the impact of decreases in both total and cortical vBMD on bone strength at the tibia may be somewhat attenuated by the small but significant increases in cortical perimeter.

### Baseline differences in trabecular microarchitecture

A surprising finding was baseline between‐group differences in trabecular number and thickness, which may be indirect evidence of early pregnancy microarchitectural changes, although without pre‐pregnancy data this is speculative. There is a paucity of literature relating to early pregnancy microarchitectural adaptations, although one historical cross‐sectional study that acquired iliac crest biopsies during early pregnancy, late pregnancy, and non‐pregnant women suggested early pregnancy trabecular microarchitecture changes might occur.^32^, ^33^ However, caution is required in comparing the current study to the histological one as the groups in that study differed significantly in age.

### Strengths and limitations

The major strength of this study is that two different pQCT modalities were used to describe bone density and microarchitecture changes during pregnancy and have been able to contrast those changes at weight‐bearing and non‐weight‐bearing sites. The vast majority of scans were obtained, graded, and analyzed by a single operator, which reduced any interoperator effects. In addition, this is the first application of HR‐pQCT imaging techniques in pregnancy and one of only a handful of studies to use conventional pQCT techniques in pregnancy. The use of HR‐pQCT allowed quantification of changes in bone microarchitecture, while pQCT allowed the scanning of cortical‐rich sites. This reduces the likelihood of any of the key findings being due to chance because both imaging techniques found broadly similar patterns of change at the distal radius and tibia despite their different scan sites and methodologies.

The limitations of this study mostly relate to the difficulties of obtaining in vivo bone densitometry data from pregnant women; we could only image the peripheral skeleton, scanning was restricted to a relatively narrow time window for assessing change, and there were no pre‐pregnancy scans. Another limitation is that we cannot determine whether any of the pregnancy‐related changes we documented are recovered postpartum or during lactation. Changes in the NPNL group for trabecular vBMD were larger than expected in this age group of women. However, there is consistency between the pQCT and HR‐pQCT measures in this study and also with DXA data, ranging from −0.46% to −1% for the hip and spine in a previous study, over a similar follow‐up time, by our group in Cambridge.^7^ Additionally the QA scans for both scanners remained within manufacturer limits. Finally, we cannot be certain that this is not a residual effect of post‐lactation‐associated bone loss as suggested by the recent Bjørnerem study.^51^ However, we selected 3 months past end of breastfeeding as an exclusion criteria based on our previous work^7^, ^50^ and it is important to note there is no consensus regarding the timeline of postpartum changes in mineralization.^52^ In addition, it is likely that the magnitude of the pregnancy‐related changes would have been greater if follow‐up scans had been obtained later in the third trimester. Furthermore, the relevance of these data to maternal bone health may depend on the extent to which subsequent mobilization of bone mineral occurs during lactation if these women breastfeed, the length and exclusivity of breastfeeding, and whether women in this age range have sufficient time to fully replete their mineral reserves before the onset of menopause.

### Conclusion

This study documents for the first time within‐pregnancy changes in both vBMD and bone microarchitecture, with changes suggestive of mineral resorption found at both the load‐bearing distal tibia in both compartments and at the non‐load‐bearing proximal radius where evidence of endosteal resorption was observed. These changes appear to take place against a backdrop of natural age‐related change in NPNL women and highlight the need for further research to determine the impact of reproduction on the maternal skeleton in the decade(s) preceding menopause. Although the magnitude of these pregnancy‐related changes in the appendicular skeleton are small, if they reflect global changes across the skeleton at large they may contribute substantially to the Ca requirements of the fetus.

## Disclosures

All authors state that they have no conflicts of interest.
